# The independence of medical ethics

**DOI:** 10.1007/s11019-018-9842-1

**Published:** 2018-05-16

**Authors:** Johan Brännmark

**Affiliations:** Culture and Society, Malmö University, Nordenskiöldsgatan 1, 205 06 Malmö, Sweden

**Keywords:** Medical ethics, Moral theory, Applied ethics, Methodology

## Abstract

This paper discusses the relation between medical ethics and general moral theory, the argument being that medical ethics is best seen as independent from general moral theory. According to this independence thesis, here explicated in terms of what is called a *disunitarian* stance, the very idea of *applied* ethics, which is often seen as underlying medical ethics (as well as many other more specific fields of ethics), is misguided. We should instead think of medical ethics as a domain-specific ethical inquiry among other domain-specific ethical inquiries. On this alternative kind of picture, such ethical inquiries should start with looking at the particularities of the domain under consideration and then proceed from there. Some possible consequences of this idea for medical ethics are then identified and discussed.

## Introduction

In a recent commentary on the state of medical ethics, Savulescu (2015, p 32) laments the loss of the “adventurousness and originality of its pioneering days” and suggests that “[m]edical ethics isn’t sufficiently philosophical”. Compared to the state of the field a couple of decades ago, there certainly seems to be something to the descriptive analysis underlying Savulescu’s claims. There has been an increased specialization and more work also has a multidisciplinary character. Most people doing medical ethics do not work at philosophy departments—they tend to be medical ethicists rather than moral philosophers who also do some medical ethics. Authors in the vein of Peter Singer and Jonathan Glover (two examplars of the more adventurous and philosophical way of doing medical ethics pointed to by Savulescu) arguably play a less significant role. As a description of how the field has evolved, this picture will not be challenged here; it will be assumed to be largely correct. The focus will instead lie on the evaluative part: is this kind of development to be regarded, at least in its general tendency, as good or bad?

An important part of the answer to the above question concerns the relation between (i) general normative ethics, i.e., philosophical theories about what is right and good (such as utilitarianism, Kantian ethics, Aristotelian ethics, etc.) and (ii) more concrete ethical reflection on moral issues arising in particular domains, like medicine. To what extent should the latter start in the former? A common working assumption in normative ethics is what might be called *unitarianism*, an idea that ethics is not domain-specific, but that there is a common ethical core, something like an implicit moral system underlying our moral deliberations and discussions, that can then be philosophically regimented into either a single highest moral principle, such as utilitarianism or Kantianism, or at least into a relatively small set of basic moral rules, such as the list of six *prima facie* duties put forward by Ross (1930) or the ten moral rules proposed by Gert (2004, 2005). Yet we could also have another working assumption, namely that what is often called common-sense morality is not systematic enough for it to be reasonable to perform this kind of two-step, where we first ascend to the highly abstract level of one or a handful of completely general moral principles and then descend to the level of concrete domains, like medicine, where we can apply these principles. Instead we would simply ascend from the morality of specific domains to principles or rules that are reasonable for those domains. It could still be the case that there are broad generalizations, e.g., “killing is wrong”, that cut across domains, but where these are simply too open to different interpretations and precisifications to be of any real use, e.g., wrongful killing in medicine and wrongful killing in war would then not be two applications of a single prohibition of killing, but two distinct moral regulations. On this kind of outlook, the developments that Savulescu points to would actually be quite reasonable, at least as general tendencies.

The argument in this paper will be in favor of what might be called a disunitarian approach to normative ethics and, *ipso facto*, to medical ethics. Although the argument here is hopefully reasonably strong, it should be recognized that methodological arguments are rarely knockdown arguments, so the main hope underlying the paper is to make the case that the possibility of doing self-consciously disunitarian medical ethics should be taken seriously. The section “Domain-specific theorizing” will identify what disunitarianism is about by relating it to other methodological choices, the “Three arguments for the disunitarian approach” section will provide three positive arguments for being a disunitarian, and the “From vertical to horizontal integration” section will delineate three kinds of integrative work that can be expected to characterize a reasonable disunitarian approach to medical ethics. It should be said that the arguments here will not directly engage with Savulescu’s position that good medical ethics is strongly dependent on general philosophical theorizing, but rather provide an alternative story on which good medical ethics is instead characterized by its independence from general and abstract ethical theorizing.

## Domain-specific theorizing

In order to clarify the idea about domain-specificity involved in the disunitarian approach advocated here, we will begin with some largely cartographical remarks, i.e., conceptually mapping it onto the terrain of some already existing methodological discussions by Beauchamp and Childress and Henry Richardson. First, however, a few words about the notion of *domains*. This is a term that will be used here to refer to sets of interconnected practices, where a practice is understood in Rawls’ sense of “any form of activity specified by a system of rules which defines offices, roles, moves, penalties, defenses, and so on, and which gives the activity its structure” (Rawls 1955, p 3n1). There is often overlap between practices and when there is a sufficient degree of such overlap, especially in terms of the offices and roles involved in the relevant practices, we will have what is here called a domain. If we take medicine for example, then there will be a number of different medical practices, but they will be characterized by certain roles typically being involved (like physicans, nurses, patients, next of kin), and so we will think of such practices as being strongly interconnected and as potentially being governed by a unified ethic. This is not to say that different actors, e.g., physician and nurses, cannot have different responsibilities, but only that it might be reasonable to look for moral principles or ethical ideals that cover all the practices in the relevant domain. What will typically characterize a disunitarian approach, then, is that one will aim at articulating such principles as being reasonable principles simply in relation to that domain. There is a question here about exactly how such domains are to be delimited, and boundaries are unlikely to be sharp, but we can actually already see a significant crystallization into domain-oriented fields: medical ethics, animal ethics, the morality of war, business ethics, climate ethics, and so on. And while the notion of “domains” is used here to explicate the disunitarian approach, the essential disunitarian tenet is not that one’s inquiries should explicitly be framed in terms of “domains” but that one does not ascend to, or refer to, the level of completely general moral theories or principles, that one’s sights are set lower. And the existence of already recognized domains (or areas or fields) seem to show that the relevant targets are there—the question is just how one will choose to engage with them.

Now, unitarians might certainly also articulate domain-specific principles, in the sense of being intended to govern a specific domain. However, to the extent that such principles are reasonable, they will be so not just because of their fit with the specific characteristics of the domain, but also because they are in line with higher-order principles that are not domain-specific. The exact principles might even be the same—the difference lies in how they are reached. Take, for example, the four-principles approach articulated by Beauchamp and Childress (2013). Both a unitarian and a disunitarian could embrace this as a reasonable ethical framework in the domain of medicine. But while for the disunitarian the question would simply be “Is this a reasonable framework given the characteristics of this domain?”, the unitarian would also ask “Is this a reasonable framework given certain overarching principles or more general common moral norms?” Even among ethicists with a clear focus on some particular domain, the difference between these two kinds of approaches is however not always noted. For instance, apart from being a highly influential textbook, *The Principles of Biomedical Ethics* also has two whole chapters devoted to assessment of moral theories and justification in ethics and Beauchamp and Childress then distinguish between and discuss three main “models of method and justification [that] operate in ethical theory and contemporary bioethics” (Beauchamp and Childress 2013, p 391): (i) *top-down models*, where we start with principles or precepts lying on a high level of generality and abstraction and we reach sound moral judgments about concrete matters by proceeding deductively and applying these to the matter at hand; (ii) *bottom-up models*, where we proceed inductively instead and reason from particular cases and where rules and principles are often seen as “derivative, rather than primary, in the order of knowledge” (Beauchamp and Childress 2013, p 398); and (iii) *integrated models*, where we work to bring general principles or rules and concrete judgments (and possibly relevant background theories as well) into a state of reflective equilibrium.

Given this tripartite schema, the disunitarian approach is however difficult to fit in, because the schema does not address the ultimate scope of one’s inquiry. In emphasizing the domain-specific, a disunitarian approach might sound as if it is a version of (ii), but not only can a domain-specific normative theory or model take many different forms, it can also be justified in different ways. What distinguishes it is that the principles or precepts that are articulated simply concern a specific domain (or that the case-oriented analogical reasoning it involves does not carry any direct implications beyond the domain in question). In actual practice, this is a methodological position that would seem to be an ill fit with a top-down approach since adherents of that kind of approach tend to start by identifying principles or rules at a high level of generality and abstraction, but at least in principle one could identify certain domain-specific principles or rules as self-evident and then see the continued work as simply bringing these principles to bear on concrete cases.

When it comes to Beauchamp and Childress themselves, they are explicit about starting in an idea of common morality, “the set of norms shared by all persons committed to morality” (Beauchamp and Childress 2013, p 417) and the considered judgments that form an important part of the reflective-equilibrium process are for them also to be part of common morality. But the reflective-equilibrium approach does not really as such presuppose this kind of common morality. Instead, medical ethicists working towards reflective equilibrium face a choice between either (i) articulating a coherent approach simply to the ethics of a specific domain, where the principles and rules articulated are in a state of reflective equilibrium in relation to the stakeholders in that domain,^1^ or (ii) articulating principles and rules which are domain-relative versions or applications of more general principles that cover all of morality. As already noted, if we take something like the four-principles framework proposed by Beauchamp and Childress, this could be arrived at from either approach; the choice of approach does however matter for how, more exactly, we would determine whether that kind of framework is reasonable or not.

Furthermore, it should be made clear that the issue of unitarianism versus disunitarianism is also distinct from the question of whether one is relying on application, balancing or specification as a way of resolving concrete ethical issues. This tripartite distinction has been explicated by Richardson (1990, 2000), albeit primarily as a way of bringing attention to what he regarded as a neglected option: specification. *Application* is the most straightforward way of proceeding, where you start with an idea like *if an action has the property F, it is wrong*, note that a particular action A has the property F, and conclude that A is wrong. If we recognize a plurality of moral rules or principles, many individual actions will however often fall under some additional rule or principle as well and a common idea is that you then resort to *balancing*: weighing the relative importance of two or more moral reasons, hopefully being able to conclude what you ought to do. The *specification* that Richardson advocates is an alternative way of handling conflicts between broad and abstract principles in concrete cases and it involves adding clarifying clauses to our initial principles. If done correctly, the principles will no longer clash in this concrete case, but instead provide specific action-guidance. There is clearly a way in which this type of theorizing will tend to be domain-specific in that the relevant specifications will tend to be concrete and limited in applicability. We might for instance have an initial principle that tells us to respect autonomy, but then add clauses that are specific to the physician-patient relation. Yet this is not a disunitarian position at root, because as Richardson points out “the model of specification presupposed that one had a theory, or at least an articulated set of norms, already in hand, and asked a question that then arises” (Richardson 2000, p 287).

The choice between unitarianism and disunitarianism is about where one thinks that the *highest-level* principles are located, about whether there are completely general principles that can be specified into domain-specific mid-level principles or rules (and then perhaps further specified as we work out the particularities of the relevant domain) or not. On both approaches there can be specification—the only difference is on which level of generality we can start the process of specification. One consequence of this difference is that while for the unitarian, one would expect all domain-specific principles or rules or virtues to still instantiate the same basic type of approach, because we have already set down a specific path, disunitarianism leaves us with an open question here. It is perfectly possible that some domains are best theorized rule-ethically and some virtue-ethically. Another difference that might exist between domains is that some are best regulated simply through substantive principles or rules, while in other domains a greater reliance on procedural rules might be reasonable—this is something that is decided based on the characteristics of the domain in question. And it is also an open question to what extent we use application, balancing or specification in handling concrete cases. For the disunitarian it could very well be the case that in working out the morality of war we should rely heavily on specification, while in medical ethics there is more room for balancing, and so on.

Irrespective of whether one frames one’s account in terms of principles/rules or virtues, the domain-specificity will mainly come through in how these are formulated. While completely general norms tend to be thought of as applying to us *qua* agents or human beings, domain-specific ones apply to us through the way in which have a role or a stake in that domain. This does not necessarily mean that in articulating rules or principles that are domain-specific, we will always explicitly include that kind of reference, but were we to fully state the rules or principles in question their domain-specificity would become clear.^2^ For instance, we could look at lying in both the domain of medicine and the domain of family life and arrive at the following two rules:

A. For physicians, in dealing with their patients, it is always wrong to lie.
B. For parents, in dealing with their children, it is permissible to lie when the child is at a level of development where it will be difficult to process the truth.

The point here is not that these are the most reasonable positions on lying to take in these two domains, but simply that it is quite possible that there are differences like this in the rules that apply to parents and physicians, that a reason like “it will be difficult to process the truth” can be morally relevant to parents, but not to physicians. At this point, it might perhaps be responded that if there is this difference between parents and physicians it must be possible to identify something about being a parent that is different from being a physician and that once we identify this further feature we could have a general principle like:

C. For any agent, who has feature F (where F is a feature that is not domain-specific, but simply one that can be exemplified in different domains), it is permissible to lie to someone for whom it will be difficult to process the truth.

It cannot however just be taken for granted, when theorizing ethics or developing ethical frameworks, that we can always identify such generic features, especially not if we are aiming at articulating what could serve as widely shared ethical frameworks (see the “Three arguments for the disunitarian approach” section). Not only does such sharing usually presuppose that the level of detail is kept down, developed ethical frameworks will always involve striking certain balances, and even if we could come to share the way a certain balance is struck, we can still vary on exactly why we find it reasonable. We could very well come to share rules and principles framed in terms of domain-specific roles like physicians and parents without agreeing on, say, the exact nature of the difference between being a parent and being a physician.

Apart from specificity in terms of principles/rules or virtues, the disunitarian approach also allows that some of the notions used in articulating relevant ethical concerns are understood differently in different domains. Take a concept like *autonomy*. It means self-determination, but just what does self-determination mean? It is quite clear that philosophical discussions about the nature of autonomy has not produced any definitive answer to this question, but rather many different conceptions of autonomy (Anderson 2014). For the disunitarian this is not necessarily a problem because the question one will ask is not “what is the most reasonable conception of autonomy and what ethical implications will it have for the domain I am looking at?”, but rather something like “which conception of autonomy has the best fit with the kinds of concerns that are relevant in the domain I am looking at?”—in the domain of medicine, it is then perfectly possible that what we primarily need is a conception that makes sense of and helps us develop consent procedures (or not, as argued by O’Neill (2002)—the important point here is just that it is a question that should be settled at the level of the domain in question).

Before turning to consider some positive reasons for adopting a disunitarian approach, it should be made clear that while the disunitarian approach involves giving up on the level of completely general principles or norms as a meaningful subject-matter for ethical inquiries, disunitarianism does not imply that one must be insular in one’s approach. It does not preclude looking at arguments or even principles/rules developed in other domains as sources of ideas. In fact, doing so might often be a good way of discovering things about one’s own domain that one would perhaps not have noticed otherwise—it is just that it would be done simply to aid one’s understanding of the domain that one is already working on.^3^ Additionally, since practices will inevitably have points of overlap between domains, one should also expect that there will be certain trans-domain moral tensions that might need to be resolved (more about this in the “From vertical to horizontal integration” section); it is just that one does not think that these will be resolved by moving up to a domain-transcending level of ethical inquiry—rather, one will work sideways, so to speak, towards greater coherence between domains.

## Three arguments for the disunitarian approach

The disunitarian approach is a general methodological position, so the idea here is not that medical ethics is an exception, but rather that in doing medical ethics as well as any other form of so-called applied ethics, there is a need to make explicit which assumption one is laboring under, whether one assumes a unitarian or disunitarian stance. It is certainly possible to not do this, at least not explicitly, but since the choice between these two approaches will have implications for the soundness of different types of arguments and different ways of conceptualizing key notions, to remain non-committal on this point comes at the cost of muddying the waters. One really should make explicit the way in which one is working and one should also have an idea about why one works that way. We will here look at three arguments for why one should be a disunitarian. Note that these are not arguments intended to show that disunitarianism is *true*, whatever that would mean, but rather that disunitarianism is more reasonable as a working assumption. The focus will lie on medical ethics, but the issue is not just one for medical ethics, so the arguments will have a broader scope.

### Lack of convergence at the top level

In their handling of general moral theories, Beauchamp and Childress take an ecumenical approach. They propose what might be called a mid-level theory that is compatible with several different top-level theories. There is certainly a pragmatic reason for such an approach, since if we look at the history of ethics, there are no indications that we are converging on a single shared general normative theory. Kantianism and utilitarianism have been around for over two centuries. Both of them have become more developed, but primarily this just means that there are now so many more versions of utilitarianism and Kantianism to choose from. Aristotelianism has come back as a serious contender, together with other forms of virtue ethics. There is rights theory, care ethics, and discourse ethics. Faced with this plethora of positions, Beauchamp and Childress opt for developing a model that is compatible with several main moral theories, but ultimately this also means that the top level of theorizing does not really do any heavy lifting in their approach. They do not frame matters in terms of unitarianism and disunitarianism, but their actual ethical framework could possibly be interpreted as a form of *functional* disunitarianism: for all practical purposes it is a domain-specific approach.

While the lack of convergence in terms of fully stated ethical theories might be dissatisfying when it comes to relating concrete issues to a highest possible top level of moral theorizing, there is also a question about what this lack of convergence might be a symptom of. Here our working assumptions really come into focus. Moral theorists standardly tend to work *as if* there more or less already is a common system of general moral principles or rules to be uncovered or made explicit, or at least that there is *something there* which structurally is similar enough to such a system so that, through moral theorizing, it can be regimented into one—like how there are rules of grammar underlying the way we use language even if most speakers of a language might not be able to fully articulate them (Gert 2005, pp 4–5). Yet this is really just an unargued assumption. It is perhaps a natural assumption to make if one has a certain idea about what the end product of moral reflection should look like, namely a unified moral theory consisting of at most a handful of principles—almost like a set of moral laws of nature—but it is still just an assumption.^4^ And given the lack of convergence in achieving that end product, one might certainly wonder if there is not something amiss with this assumption. Even if we accept that there are regularities in our moral judgments which moral theorists can attempt to regiment into some form of rules or principles, it is a perfectly open question on which level of generality these regularities lie. They could certainly be absolutely general, but they could just as well lie at the level of different concrete practices in which we regularly participate, or at the level of certain areas or domains of life consisting of clusters of such practices.

General rules like “Do not kill,” “Do not lie,” and “Do not steal” are clearly part of everyday moral discourse, but if you scratch the surface these are not really accurate generalizations of our moral responses. There are exceptions to all of them so if there are accurate generalizations to be articulated about these types of actions, they must be much more detailed. Is there then a single detailed and fully specified rule against killing that applies across all areas of life and that we can uncover? Or a series of different such rules with respect to different domains or areas of life? It is surely possible that there is one such rule against killing that applies equally to the medical domain, to the arena of war, and to the workings of the criminal justice system, but it is also possible that there are different (albeit partly similar) rules against killing in different domains. And in the latter case, to assume that one should work towards a single unified prohibition of killing, from which judgments about killing in different domains can then be derived, is only likely to impede progress. So there is a choice to be made here and it is not obvious that the standard approach, unitarianism as an implicit working assumption, is the most reasonable one, especially given the lack of convergence that we have seen throughout a very long history of ethics. It is a potentially unnecessary detour into abstraction, one that philosophers certainly often take, but perhaps mostly because it is at abstraction that they excel. In fact, agreement in morals often seems to be more readily achievable at the level of mid-level theorizing anyway. So, given its poor historical track record, why not just give up on the traditional top level of moral theorizing?

### Reliance on intuitions

That moral theorizing relies on testing ideas against our intuitive responses should be evident to anyone who has studied normative ethics. Such tests are often done by carefully constructing thought experiments where irrelevant factors are weeded out, so that we can then distill the directly relevant responses that we can use in order to gradually move towards more refined moral theories. But what reason do we have to rely on specific intuitions as guiding us towards something better? Presumably we can have intuitions for all sorts of reasons and some intuitions might just be prejudiced or confused. This is really a challenge that all normative theorizing faces, as long as it relies on our intuitive responses. The classic Rawlsian answer that we should rely on our *considered judgments* (Rawls 1971, p 42) is not altogether satisfactory, because the bare fact that something survives an initial process of reflection does not mean that it is enlightened, especially given our well-documented capacity for motivated reasoning (Kunda 1990). One possibility here is to look at the extent to which some intuitions can be explained away, such as by putting them into their historical context. For instance, Peter Singer argues that many of our moral intuitions have been “shaped by our parents and our teachers, who were either themselves believers, or were shaped by those who were” (Singer 2005, p 345), the point being that we need to free ourselves from this Christian influence in order to formulate a rational secular ethics. The reasoning behind this kind of suggestion seems problematic, however. None of our moral convictions have appeared *ex nihilo*, so the bare fact that we can provide historical context for certain intuitions cannot decide whether they are reasonable or not. Our moral intuitions also seem to differ on many issues, so it is not clear, even to begin with, if there really is some monolithic common-sense morality that history, or evolution for that matter, has dumped in our laps.

An alternative strategy would be to instead focus on the quality of the concrete experience underlying our intuitive responses. If we start in the psychology of decision-making, Kahneman and Klein (2009), coming from two very different directions in psychological research, have suggested two conditions on a relevant environment for skilled intuitive responses to develop among (at least some) decision-makers there. First, decision-makers need to be in a *high-validity environment*, i.e., there must be regularities which are available to us in the form of reliable cues so that we can recognize patterns; second, the environment must *provide adequate opportunities for practice*, i.e., in order to develop skilled intuitions we must not merely be placed in a high-validity environment, we must be able to make judgments and decisions in it and receive feedback from the environment about the quality of our judgments and decisions. Note that these are necessary rather than sufficient conditions for skilled intuitive responses to emerge, but if there is a question about which intuitions that we should put our trust in, the fact that some intuitions have this kind of background would at least seem to give some weight to them. One thing that will characterize these skilled intuitions, however, is that they will tend to be domain-specific because the kinds of environments where we can get relatively consistent and regular feedback will tend to be ones that have regularity, structure, and stability to them. *Medicine* is arguably such a domain, *human agency* is not—the latter covers an enormously varied range of decision-making environments. There is certainly a question here about exactly on which level we best identify relevant such environments, but the disunitarian approach is clear: they should be relatively concrete and specific.

In contrast, traditional moral theorists have by and large not accorded actually having relevant experience much weight in discriminating among different intuitive responses. It is difficult to say why this is the case. Part of the explanation might be that many of the thought experiments that are utilized are so heavily artificial that no-one would have experience of situations like that anyway. But it should perhaps also be kept in mind that an approach that privileged actual experience would often not privilege the intuitions held by philosophers, but rather point instead to the intuitions of different kinds of practitioners or stakeholders as more valuable input. If we look more broadly at different ways in which we learn to act well, it is actually relatively common that we learn how to handle specific practices or domains well rather than achieving a form of universal competence that we then apply to particular domains. And if competence is rarely (if ever) universal but something that we normally find in relation to specific practices or with respect to specific domains, then why should moral competence just be assumed to be a kind of universal competence? People clearly seem to be able to be morally upstanding in one area, while morally deficient in another, so the same should go for our moral intuitions: they can be sharp in one area and blunt in another. If we want to be able to work with skilled intuitions, we accordingly seem to be driven away from the abstract and completely general and towards concrete areas of solid experience instead.

### The publicity of moral norms

Most normative theorists tend to rely on some kind of reflective-equilibrium approach and in developing that approach Rawls (1974) also argued for what he called “the independence of moral theory,” the idea being that moral theory was independent from other branches of philosophy. In striving for reflective equilibrium between principles and judgments one does not need to presuppose particular views in metaphysics, philosophy of language, and epistemology. For instance, the question of whether a particular moral theory is *true* can be set aside. At least for Rawls, this kind of independence did however not mean that moral theorizing is a private enterprise where a theorist simply tries to bring his or her own principles and judgments into a state of reflective equilibrium. Moral questions are shared questions and so moral principles need to be constructed so that they can be shared principles.^5^ For theoreticians this poses a problem for while it is relatively easy to formulate coherent moral theories, there is no guarantee that these theories will have any realistic chance of becoming shared moral frameworks.

There are basically two possible conclusions one can draw from this observation. One is to sharply distinguish between two steps, (i) the theoretical articulation of principles and (ii) the public acceptance of principles, and to then as a theoretician focus on the former. This would also mean that in deciding between different ways of making sets of principles and judgments coherent, the deciding factors should reasonably be the extent to which different theories possess certain theoretical virtues, such as simplicity and systematicity. While this strategy is understandable, since it lets theoreticians focus on what they do best, it is also problematic in that there is an obvious risk that the pursuit of such theoretical virtues will lead to a level of abstraction that makes a theory less likely to be a good fit with the intricacies of everyday life—or can even be properly understood by most people. And do we really want that much separation between theory and practice in ethics? There is also a clear risk that in pursuing theoretical virtues one will arrive at an extremist theory—not that simplicity automatically spells extremism, but there is arguably a link between extremism and the idea that *it is all so simple, really*. Are the methods of ethics robust enough so that we can trust them to steer us clear of misguided moral extremism?

The alternative lesson one might draw is that already in the articulation of our basic principles we need to think in terms of the workability of those principles when released into the wild (so to speak). This is clearly the way Rawls himself approaches his principles of justice, e.g., in his reliance on what Norman Daniels has identified as background theories: “a theory of the person, a theory of procedural justice, general social theory, and a theory of the role of morality in society (including the ideal of a well-ordered society)” (Daniels 1979, p 258). This focus on the kind of society or community for which the principles are supposed to be guiding principles does not mean that theoretical virtues do not matter in constructing theories, but simply that there are also practical virtues to consider when developing moral and political theories. What is the job of principles? To aid in moral decision-making is an obvious answer. But if we think that principles are something that should ideally be more or less shared in a society or a community, then what is of interest is not just potentially assisting the discrete decision-making of individuals, but the extent to which such principles can facilitate productive dialogue on moral and political issues. They should help us in communicating about relevant issues and to reason together in resolving them.

If one opts for this second approach (which arguably one should), the choice between a unitarian or a disunitarian approach comes down to this: on which level of analysis is the workability of principles or models of sound ethical deliberation best assessed? Workable principles need to be principles that we can relate to, ones that speak to our moral experience. As already noted, completely general and abstract principles are usually thought to be ones that we are to relate to simply *qua* agents or human beings, whereas if we focus on domain-specific principles, these will tend to be ones that we relate to in some more specific capacity, such as physicians, nurses, patients or citizens. At least for most non-philosophers, this is probably a stronger form of relatability. It is through our more specific capacities that we become stakeholders in relation to concrete ethical issues. Different domains also tend to be characterized by different configurations of stakeholders. Articulating principles that directly address this fact should facilitate communication in a way that highly general and abstract principles might not. Additionally, the latter kind of principles will also tend to be characterized by a stronger degree of openness; it will be more unclear just what implications they have. This makes it likely that people will treat them with some wariness, not being sure about whether to fully commit to them since it is not entirely clear what you are then buying into. To the extent that there is this kind of uncertainty it seems likely that communication about concrete moral issues will be better facilitated by an approach that simply abandons the level of completely general and highly abstract principles and focuses directly on the relatively concrete and specific instead.

## From vertical to horizontal integration

The extent to which the above arguments, if accepted, have any substantial implications for the way medical ethics is done depends on how one is already doing medical ethics. Disunitarianism could simply be a meta-story confirming that one was doing it more or less right all along. As already pointed out, medical ethics is often practiced more as a distinct subject than a subdivision to moral philosophy. It should also be kept in mind that disunitarianism is primarily a *negative* position: it tells us what not to do, but that still leaves a lot open. There are many different ways in which one can be a disunitarian. Just as there are many different ways of being a unitarian.

While it is not a necessary consequence of adopting a disunitarian position, there is however still a certain shift of focus that such a move readily invites and in this last section, we will look into what this might involve. Anyone engaged in a systematic inquiry into moral rules and principles, or virtues for that matter, will tend to look at ways of integrating different strands of thought into a more strongly coherent picture. For a unitarian, the focus will typically lie on what might be called *vertical integration*: the extent to which we can achieve coherence between different levels of abstraction and generality. The underlying idea, then, is presumably that normative beliefs can first be grouped in terms of the level of generality and abstraction at which they lie and that our process of reflection is primarily about going back and forth between different levels. But in understanding the kind of reflective equilibrium that we are aiming at, we could also be thinking in terms of what might be called *horizontal integration* as well, where an important part of reflection instead involves going back and forth between different clusters of ideas that lie at more or less the same level of generality and abstraction.

Although not explicitly articulated in these terms, one kind of horizontal integration is an important concern for Rawls in his later work (Rawls 1993), with its emphasis on the notion of an *overlapping consensus* and achieving a *wide* and *general* reflective equilibrium. The starting-point for Rawls is then what he calls *the fact of reasonable pluralism*, the fact that in modern complex societies we cannot even expect all reasonable people to be in complete moral agreement. We must expect and accept diversity, but at the same time also try to articulate principles that we can share with respect to the core institutions that we share. While not necessarily drawing the same conclusions from this kind of observation, a similar argument has been made with respect to medical ethics by Engelhardt (1996, 2015): moral diversity must be taken as a starting-point, not in the sense that it is something that we start with and then aim to overcome, but rather that we need to recognize it as the inevitable context of inquiries into medical ethics (or any other domain of ethics for that matter).^6^

The Rawlsian position here is that we can have diversity and still some commonality and that moral and political philosophy can play a part in facilitating the articulation of principles that we can share on a societal level. It is not necessarily the case that a disunitarian must take this approach, but with diversity comes the possibility of conflicts between different norms and values. And while the unitarian can hold out hope of transcending and ultimately resolving such conflicts by moving to the highest level of abstraction and generality, the most natural way for the disunitarian to address such issues is to look at different possibilities of horizontal integration of interests, ideas, and principles, where something like an overlapping consensus is a reasonable way of achieving this.^7^ Now, someone like Rawls primarily understands diversity in terms of cultural pluralism, but there are at least three more specific forms of horizontal integration that might be important for medical ethics:

### Integration between types of stakeholders

While in some domains, say, the ethics of friendship, there is a relatively high degree of symmetry between the stakeholders, decision-making in the medical domain is characterized by significant asymmetries between the stakeholders, for instance in where their areas of expertise lie (and where they might potentially also have formed highly relevant moral intuitions). There are at least three important types of stakeholders to consider. We have physicians and nurses, who of course have the medical expertise. We have patients and their next of kin, who are experts on their own lives and the concrete impact that different effects of treatments might have on them. But we also have health-care policymakers and administrators, who might not be in the room when specific decisions are made, but who are still to a large extent responsible for setting the parameters for those decisions, and who are (hopefully) experts on large-scale prioritizations and implementation processes. This kind of relatively concrete identifications of different types of stakeholders primarily makes sense on the level of domains.^8^ Now, as already pointed out, the degree to which there is even a need for explicit principles can vary between different domains. For the disunitarian it is not *one size fits all*. Some domains might be relatively informal and personal, where we can keep things implicit and something like a particularist approach might even be appropriate. But since the medical domain is a central societal institution where important parts of its workings are subject to political decisions, it seems appropriate that it is characterized by relatively explicitly articulated values and principles, especially since there is a need to facilitate communication between stakeholders of quite different kinds.

### Integration between morality, politics, and law

One possible worry with respect to disunitarianism is that it involves accepting a fragmentation of the field of ethics and will, because of this, complicate rather than facilitate communication. But while there is a certain type of fragmentation that is inherent in the disunitarian approach, there is also a certain type of fragmentation that the unitarian approach tends to underpin, namely that morality, politics, and law are at the end of the day distinct theoretical domains. Since a main concern for unitarians will be integration with general moral theory, it seems likely that other kinds of integration, already at the stage of inquiry and not just at the stage of application, will take the backseat. Especially with respect to medical ethics, this would be an unhappy state of affairs since medical decision-making is so clearly located at an intersection between morality, politics, and law. This might not be a characteristic of all domains (e.g., if we take something like the ethics of friendship instead, law and politics might not be all that deeply enmeshed with the intricacies of what makes a good friend), but if we look at contemporary medical ethics, the relevance of this kind of horizontal integration can be seen already in how it has developed. For instance, if we look at the role of law, the development of informed consent procedures arguably took place precisely as a form of horizontal integration between existing negligence law and moral ideas about responsibility and self-determination (cf. Katz 1994). And in terms of politics, questions of distributive justice are clearly central to the provision of health care and the practice of medicine in contemporary societies; these are not concerns that are external to medical ethics. For the disunitarian this kind of integration will typically look less like a lack of purity in our ethical thought and more like starting in the actual characteristics of the domain in question. Of course, unitarians can certainly also be concerned with law and politics, but what disunitarianism points to is a conception of medical ethics where morality, politics, and law are more strongly integrated than just being ultimately relevant to each other, i.e., not just as something that you look at in order to apply more general principles, but as something that you take in already when articulating your basic principles.

### Integration between domains

As already pointed out, even if we can identify certain reasonably large-scale interconnected complexes of human practices regulated by certain norms and values, such domains will never be completely separate from each other. There are arguably *sharp enough* boundaries for medical ethics to be recognizably distinct from environmental ethics, for the morality of war to be distinct from business ethics, and so on. But there will always be areas of overlap between different domains. For instance, while medical ethics and business ethics are distinct, the regulation of pharmaceutical companies and intellectual property rights certainly has bearing on access to medicines (cf. Grover et al. 2012). Now, the important point here is not about any particular such issue, but simply to make clear that in order to develop reasonable approaches to problems located in such overlaps between domains does not presuppose that we should move up to a higher level of abstraction and then derive a solution from there, which would presumably be our main method of resolving conflicts if we were primarily oriented towards vertical integration and traditional moral theorizing. Instead, if we are interested in the workability of different possible solutions, what we need to do is to look at the particularities of both the relevant domains, because it is at that level that we will be able to locate viable compromises. To the extent that we seek greater coherence between domains, which there might be practical reasons for doing, we will (again) be seeking a horizontal form of integration.^9^

A workable solution to a problem like the regulation of pharmaceutical companies will need to make sense both from the side of business practices and the practice of medicine. This is not the same as saying that there should be equal weight accorded to all interests that are involved, only that the solutions that we come up with need to make sense from the respective perspectives of the relevant parties and that resolutions of conflicts will be like reasonably stable peace treaties, rather than like pronouncements from some outside arbiter. Now, the unitarian moral theorist is at this point likely to say that what we want is precisely to be able to turn to something like an outside arbiter and that this is why we should be unitarians. But the problem is just that there are always several different unitarian moral theories, which means that different stakeholders are likely to turn to different abstract and general moral theories, depending on which such theory that supports their initial position. We have then only moved our disagreement up a level—and not just that, but to a level where it is more difficult to reach a compromise.

A main difference here will be one of mindset. While unitarians will tend to approach issues like this in terms of trying to *discover* what the right answer is, disunitarians will approach them in terms of trying to *construct* a solution that can reasonably be shared among the relevant stakeholders. Apart from the fact that this might in the end be an unavoidable approach anyway, at least if we are seeking to regulate shared societal institutions, it should be pointed out that, given that we stress the public character of moral norms and how we relate to moral issues as stakeholders of some kind (rather than simply *qua* agents), the main thrust of systematic moral reasoning will not so much be about principles or rules that can be straightforwardly used as a decision procedure in individual cases, but as providing a basis for moral and political discussions in general and the shared decision-making of patients and physicians in particular (in the case of medical ethics). If an ethical framework is to play this kind of role it is very difficult to see how it can have that character without striking certain balances between different entry-points into such dialogues. But then we should often also expect our ethical frameworks not to straightforwardly solve or resolve difficult issues, but rather to facilitate our joint handling of them.

## Concluding remarks

This paper has had three main sections and three primary goals: (i) to explain what characterizes the disunitarian position, (ii) to provide some arguments for having disunitarianism as one’s working assumption, and (iii) to draw out some possible consequences that making this assumption can be expected to have when working on medical ethics. Especially with respect to (ii) and (iii) there remains much to be done, but the ambition here is not to have said the final word on these matters, far from it, but simply to have provided some impetus to further discussion of them. Given the kind of picture drawn by Savulescu, much of contemporary medical ethics, including how it is institutionally organized, can be read almost as if there already were disunitarian ideas behind it. The argument here can accordingly be understood as defending this development, or at least its general tendency. There has not been any room for looking closely at possible arguments in the other direction, although it should also be recognized that there is general a lack of well-developed such arguments since unitarian moral theorists tend to simply take unitarianism for granted. Hopefully this paper has at the very least contributed towards clarifying a potentially important methodological choice, namely between unitarianism and disunitarianism, and then the explication of a more developed defense of unitarianism can be left to the unitarians.

## Footnotes

1 Disunitarianism is an approach for ethicists, or possibly other people seeking to articulate certain moral principles (or rules or virtues) that they can share. This is perfectly compatible with many of the relevant stakeholders believing in some form of common morality or higher-order principles, just as some of them might have religious reasons for taking certain ethical stances, and others not. Reasonable domain-specific principles will presumably be the object of an *overlapping consensus* (Rawls 1993, pp 144–150) on the level of that particular domain, i.e., a state of affairs where we agree on certain principles but might have partly different reasons for why we find these principles reasonable.

2 While this kind of approach can be expected to emphasize role-relative duties, it does not imply that duties are always role-relative. It is perfectly possible that some duties, even though domain-specific, are held by all people holding a stake in that domain—the point is just that we hold them *qua* such stakeholders rather than simply *qua* moral agents (although being a moral agent is certainly a necessary condition for holding any duties to begin with). Neither does this approach imply that as individuals we should understand ourselves simply as the sum of the stakeholder positions that we occupy, if for no other reason that we always occupy a range of such positions and will still have to figure out who we are as wholes. The point is just that given a disunitarian stance, the kind of results that ethicists will produce, at least to the extent that they concern us, will address us in some specific respect(s), e.g., as physicians or nurses or as patients or next of kin (in the case of medical ethics). It should certainly then be admitted that a disunitarian approach will not provide much of a guide to becoming an integrated moral subject, but arguably most people tend to manage that anyway, without consulting completely general ethical theories.

3 One potential worry here is that practitioners in a given domain might share problematic ideas, such as physicians believing that lying for paternalistic reasons is relatively unproblematic, and one potential advantage that a unitarian approach could then have is that it allows us to bring in perspectives from outside of the domain in question, which can help us expose problematic reasoning. With respect to this particular example, however, an important part of the problem with physicians traditionally being overly paternalistic was arguably that medical ethics tended to be seen as a professional code for physicians and by physicians, rather than as a set of shared norms in a specific domain that really involves several types of stakeholders, including patients. As suggested later on in this paper, the disunitarian approach emphasizes bringing the perspectives of different stakeholders in the domain to bear on the norms under consideration. This is probably a much more important corrective to self-serving reasoning by a specific group, especially since such reasoning often takes the form of appeals to lofty principles anyway.

4 Griffin (2015, p 119) has made a similar point in terms of how moral philosophers has, since the Enlightenment, simply assumed a conception of ethical thought modelled on Newtonian mechanics, aiming at set of abstract master principles that explicate, so to speak, the fundamental moral forces and their interactions, but where very little thought has gone into considering why this would be a reasonable approach. While rejecting the kind of grand theorizing that is a hallmark of unitarianism, Griffin does not adopt the kind of disunitarian position advocated here, but instead suggests that moral philosophy can mainly seek to make a real contribution by helping, with an eye to the relevant practicalities, to make notions like justice, fairness, equality, and human rights more determinate.

5 This public character of moral norms is also an important part of how a unitarian like Gert explicates common morality (Gert 2005, pp 11–13).

6 This kind of point might be interpreted in two main ways, either as an expression of some substantive moral meta-norm or in terms of a methodological (or perhaps epistemic) stance. For the disunitarian it is only the latter interpretation that makes sense (since the former would presumably involve domain-transcending moral norms).

7 Achieving a domain-specific overlapping consensus is perfectly compatible with some people who are parties to that consensus holding strongly unitarian views. If we are to create shared ethical frameworks, however, one requirement of reasonableness that needs to be in place will be *non-fundamentalism*: that participants in that dialogue need to recognize that they cannot expect the final product to simply reiterate the moral code they themselves started out with; the goal is rather to articulate a framework that can be acceptable, as reasonable enough, from different ethical perspectives.

8 It should be noted that since domains are complexes of practices, it is perfectly possible (indeed, it should be expected) that different practices within the same domain will feature different configurations of stakeholders, e.g., in the domain of medicine, practices surrounding organ donations will typically differ from practices surrounding medical treatment of minors, practices surrounding childbirth will differ from practices surrounding intensive care, and so on. And even if we formulate a general framework for the domain as a whole, it is quite possible that in applying this framework, part of that process of application will involve integrating perspectives from practice-specific configurations of stakeholders. (Incidentally, while most of the discussion here has assumed what might be called a *moderate* disunitarianism, one according to which ethical inquiries that cover entire domains make sense, disunitarianism is at root a negative position, giving up on the completely general level. This means that one could in principle also embrace a *radical* disunitarianism, which would involve rejecting even the level of domains as meaningful and simply make narrow *practice-specific* ethical inquiries instead. This is however a possibility that has here been set aside.)

9 Note that this kind of horizontal integration is different from the one involved in a wide and general reflective equilibrium, or an overlapping consensus, *á la* Rawls. The latter involves integration between the set of principles that form the object of consensus and the comprehensive ethical outlooks of those that are parties to the consensus. But a disunitarian approach would presumably leave us with a range of overlapping consensuses, e.g., one in the domain of medical ethics and another in the domain of business ethics, and there is then the possibility of seeking horizontal integration, in the sense of greater coherence, between different such sets of ethical principles (or whatever forms the objects of these overlapping consensuses take). But as such, disunitarianism is quiet on whether we also need to seek greater trans-domain coherence, (if so) how important it is, and how it is best accomplished. Other methodological values will have to decide this—for instance, is it a desideratum on ethical inquiries that they yield results that enable us to handle moral conflicts that we tend to face or is it enough that they just provide us with an understanding of what the basic moral principles are? The main point here, however, is simply that in facing conflicts between concerns belonging to different domains, we are not merely left with a choice between either seeking solutions to these by transcending to a domain-neutral level or just having to live with such conflicts. We can seek greater trans-domain coherence without introducing a domain-transcending level of moral principles.
